# Developing an environmental adaptation framework for older migrants in Guangzhou, China

**DOI:** 10.3389/fpubh.2026.1756061

**Published:** 2026-04-09

**Authors:** Qian Tang, Nikmatul Adha Nordin, Muhammad Azzam Ismail

**Affiliations:** 1 Department of Urban and Regional Planning, Faculty of Built Environment, Universiti Malaya, Kuala Lumpur, Malaysia; 2 Healthy and Sustainable Built Environment Research Center, College of Architecture, Art and Design, Ajman University, Ajman, United Arab Emirates

**Keywords:** older migrants, environmental adaptation framework, Guangzhou, life satisfaction, perceived living environment

## Abstract

**Introduction:**

The sharp rise in older migrants presents significant challenges to social integration in urban areas, particularly in major migration hubs like Guangzhou. Research on older migrants has typically been addressed within the broader contexts of aging and migration studies, but it remains underexplored as a distinct and focused area of study. This research aims to identify the contributing factors of the perceived living environment that influence the life satisfaction of rural–urban older migrants, and to explore the impact pathways through environmental adaptation in the Chinese context, using Guangzhou as a case study.

**Methods:**

The Perceived Living Environment Scale was used to assess the physical and social conditions of the environment, while the Environmental Adaptation Scale was developed to evaluate perceived fit among urban migrants across eight dimensions. A total of 385 older migrants participated in this survey.

**Results:**

The study proposes an environmental adaptation framework that outlines the pathways through which the living environment affects the life satisfaction of older migrants in Guangzhou.

**Discussion:**

This framework offers theoretical insights into the relationships between housing, urban infrastructure, social integration, and well-being, while also serves as a practical guide for policymakers in creating age-inclusive environments that improve the quality of life for older migrants.

## 1 Introduction

As the largest developing country, China’s rapid urbanization has prompted a sustained large-scale migration of rural populations to urban areas. Catalyzed by the 35-year implementation of the one-child policy (1), a significant social phenomenon has emerged wherein many left-behind older parents in rural areas relocate to cities with their children. The migrant population aged 60 and above had reached 18 million by 2020, with a continuing upward trend (2). Against the backdrop of the growing number of older migrants—a tangible challenge to social integration in urban China—issues affecting the life satisfaction of these older migrants are attracting increasing scholarly and policy attention.

Existing research indicates that life satisfaction among older adults is associated with a variety of factors. In addition to physical health and functional ability (3, 4), social networks (5, 6), living arrangements (7, 8) and economic conditions (9, 10), the quality of community services and the living environment also serve as strong predictors of life satisfaction among older people (11, 12). Yan et al. (13) conducted an empirical study examining the satisfaction levels of older residents across different community types. Their research confirmed that the neighborhood environment, alongside the provision of senior services provided by communities, health and income, and levels of social support, exert significant influence across diverse community settings. A significant positive relationship was identified by Gao et al. (14) between the perceived quality of the built environment and health-related quality of life. Their research which was conducted across different neighborhood contexts demonstrated that favorable perceptions of environmental dimensions such as diversity, safety, and esthetics were related to better physical and mental well-being. However, while the literature on the impact of individual and residential environmental factors on residents’ satisfaction or health continues to expand, existing research tends to focus on determinants within a single dimension or on isolated factors across different dimensions. There has been little effort to integrate multidisciplinary, multi-level factors influencing life satisfaction, and empirical studies concerning older residents remain exceedingly rare.

Older migrants, emerging as a consequence of China’s urbanization and demographic shifts under the one-child policy^1^, primarily follow two distinct patterns of internal late-life voluntary and involuntary migration. Voluntary rural–urban migration among the older in China is primarily driven by familial factors, particularly adult children’s relocation, resulting in the “Older Migrants Following Children” phenomenon. Unlike earlier generations, contemporary Chinese seniors often pursue emotional support by residing with their adult offspring while simultaneously providing care for their grandchildren (15). Enhanced healthcare access and supportive policies, such as urban *hukou* schemes which are China’s household registration system, further motivate this mobility (16). Involuntary older migration in rural China often results from urban renewal and rural demolition and relocation. As urbanization advances, rural lands are frequently expropriated for infrastructure and real estate projects (17). In response, the government has established resettlement communities on urban fringes, providing housing or financial compensation, thereby displacing significant numbers of older adults to peri-urban areas.

However, due to difficulties in adapting to new urban environments and factors such as cultural disparities, migrants generally experience lower levels of well-being than native residents (18). This phenomenon is particularly pronounced among older migrants who have resided in rural areas for many years. Menec et al. (19) suggest that older local individuals residing in a multi-ethnic urban neighborhood may perceive their community distinctly compared to older adults living in a less ethnically varied town situated in a predominantly rural location. Furthermore, factors such as language barriers, discrimination, and cultural expectations can exacerbate feelings of isolation and mental health issues among older migrants (20, 21). It is evident that older migrants face dual pressures in adapting to their physical environment and engaging with their social neighborhood. Differences in environmental adaptation capabilities can lead to disparities in the wellbeing of migrants and local residents, hindering effective social integration and impacting the harmony of the city as a whole. Understanding the diverse backgrounds of older migrants and their self-assessment of environmental adaptation is crucial for developing targeted solutions. Yet, the impact of rural-to-urban migrants’ satisfaction with their living environment and their degree of environmental adaptation on their overall life satisfaction has scarcely been researched.

To address these knowledge gaps, this research employs older individuals who have relocated from rural areas to urban communities in Guangzhou, China as the participants. First, this study identifies the features and levels of life satisfaction and environmental adaptability of older migrants with different sociodemographic characteristics in Guangzhou, China. Second, it seeks to explore how perceived living environments correlate with their life satisfaction, and to examine the mediating role of environmental adaptation within diverse community contexts as a core component of social integration. This study examines the influencing factors underlying the life satisfaction of older migrants in different types of communities within China’s major urban areas through a comprehensive assessment of individual factors (11 aspects) and living environment factors (encompassing 5 dimensions of the physical environment and 3 dimensions of the social environment). Finally, this study establishes an environmental adaptation framework illustrating the pathways through which the living environment influences life satisfaction among older migrants in Guangzhou.

## 2 Literature review

### 2.1 Perceived living environment and its association with life satisfaction

A ‘living environment’ is typically characterized by its built, natural, and social attributes, which collectively impact the quality of life and health outcomes for its residents. This concept is fundamental to how human experiences, habits, and general well-being are shaped (22). The concept of ‘age-friendly community’ has gained tremendous global attention in the field of research on the living environments of older people. Yu et al. (23) proposed an evaluation system for age-friendly communities in China that encompasses two macro-level dimensions of community environments: the physical aspects and the social aspects. It stands as one of the most comprehensive indicator systems currently available, and is well-suited for research into diverse metrics assessing the environmental quality of residential areas or communities in the Chinese context.

Previous studies indicate that satisfaction with the living environment is positively correlated with overall life satisfaction, as evidenced by studies conducted in diverse geographical locations. Khorasgani et al. (24) found a significant positive correlation between the physical and social variables of the living environment and life satisfaction among residents in Rasht City, Iran. Similarly, Ma et al. (25) reported comparable findings in Beijing, China, reinforcing the notion that both the physical attributes of the environment and social interactions therein contribute to residents’ life satisfaction. This aligns with findings from Bayar and Türkoğlu (26), who noted that urban environments, particularly those designed with older adults in mind, significantly enhance life satisfaction by providing accessibility to essential services and fostering social interactions.

### 2.2 Environmental adaptation and life satisfaction

Adaptability is commonly characterized as the capacity to adjust to novel circumstances, and its influence on happiness has been extensively studied. Zhou and Lin (27) emphasize that persons with greater adaptability can effectively employ their psychological resources to maneuver through changing surroundings, resulting in increased life satisfaction. This conclusion is supported by Chee (28), who observes that social adaptation has a substantial correlation with happiness in Malaysian young adults, indicating that social support enhances this association. Chen et al. (29) similarly discovered that cross-cultural social adaptation favorably affects life satisfaction among Chinese medical aid team members in Africa, suggesting that adaptability is essential in varied cultural environments.

The environmental context strongly affects life satisfaction. Rajani et al. (30) examine how environmental elements, including severe temperatures, can negatively impact well-being, especially in people unused to these conditions. The research by Timlin et al. (31) underscores the significance of perceived environmental elements in influencing life happiness during climate change. The relationship between environmental factors and individual adaptation is essential, as those who can adjust to their environments generally report greater life satisfaction. Furthermore, the significance of social capital and community support in improving life satisfaction via flexibility is paramount. For example, the research conducted by Yan et al. (32) demonstrates that social adaptability is a crucial determinant of human capital levels, which subsequently influences life satisfaction. This underscores the interdependence of social networks and adaptation in promoting well-being.

Compared to younger individuals, older adults appear to be more vulnerable to the impact of changes in their living environment and associated social relationships, and find it more challenging to transition to new living arrangements or social circles (33, 34). As older adults become detached from the workplace, most find their social interactions increasingly confined to family and neighborhood relationships. With more time spent within the community environment, perceptions of and integration into the living environment become increasingly linked to older adults’ life satisfaction.

### 2.3 Conceptual framework

Investigating the influence of older migrants’ living environments on life satisfaction is complex; it is essential to investigate not only the direct relationship between these variables but also the indirect effects of other factors on their interaction. Consequently, the ‘Person Environment Fit (P-E Fit)’ Model serves as a guiding framework.

The P-E Fit model originated from Kurt Lewin’s field theory, posited that behavior is a function of both the individual and their environment (35). This theoretical framework emphasized the significance of situational factors in influencing individual results and established the premise for perceiving fit as a dynamic interaction rather than a fixed attribute. Edwards et al. (36) subsequently proposed atomic, molecular and molar methodologies within the overarching framework for P-E fit research. The methodologies are situated within the domain defined as the phenomenology of person-environment (P-E) fit, which pertains to the individual’s subjective experience, the environment, P-E inconsistencies, and P-E fit. While traditional P-E Fit models focus on the congruence between employees and work environments, this study extends the framework to the domain of Environmental Gerontology, specifically examining the fit between older migrants and high-density urban residential environments.

**Figure 1:**
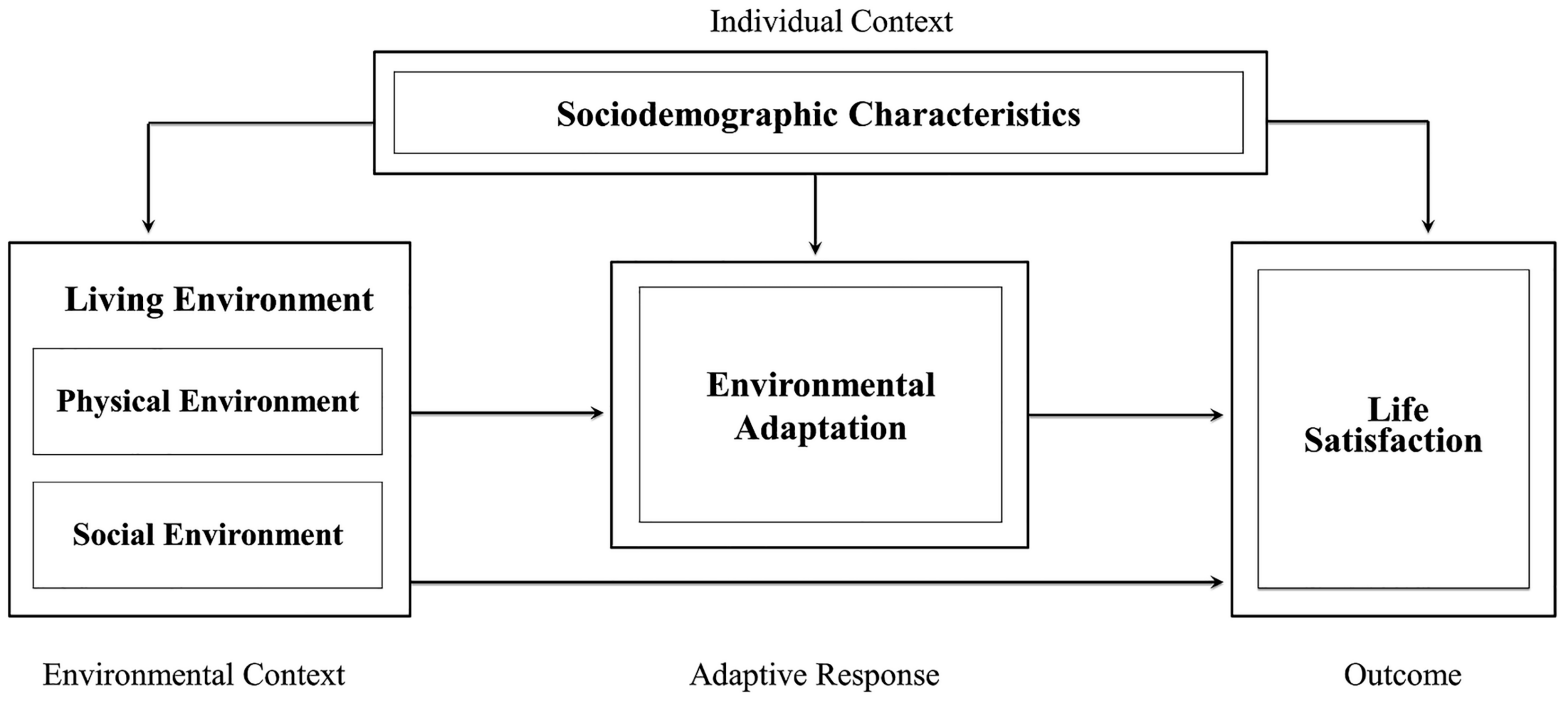
Conceptual model of the association between perceived living environment and life satisfaction among older migrants and environmental adaptation mediates the association.

Based on the aforementioned theoretical framework, this study constructed the conceptual model depicted in Figure 1. This framework, grounded in the logical components of individual context, environmental context, adaptive responses, and outcome, aims to explore the association between living environment and life satisfaction among older migrants and to examine how environmental adaptation mediates the association when main sociodemographic characteristics are controlled.

## 3 Materials and methods

### 3.1 Data collected and sample

#### 3.1.1 Study area and community samples

##### 3.1.1.1 Rationale for city selection

Guangzhou, situated in the central-southern part of this province at the northern edge of the Pearl River Delta, serves as a vital national central city, an international commercial hub and a comprehensive transport nexus in China. The city of Guangzhou in Guangdong Province was selected as the study area for several reasons. First, Guangzhou, a highly developed city in China, exemplifies the nation’s urbanization advancement and serves as a primary destination for rural–urban migration. Secondly, Guangdong Province possesses the highest population in China, whereas Guangzhou City has the greatest resident population within Guangdong Province (37). The most populated city serves as a substantial source of research samples and typically yields a diversified sample due to its aggregation of individuals from various geographic, cultural, and socioeconomic backgrounds. What’ s more, Guangzhou’s swift urban development has revealed numerous strengths and challenges in the planning of the living environment, and utilizing it as a case study can yield progressive guiding recommendations for other slower-developing second- and third-tier cities.

##### 3.1.1.2 Sampling frame and community type selection

To ensure that the participants’ living environments were relatively evenly distributed across the city, the selection of sample communities preceded participant recruitment. Based on the primary housing typologies published by the Guangzhou Municipal Housing and Urban–Rural Development Bureau^2^ and statistical data from the Official Website of Guangzhou City (38), this study identified the top five community types where older adults in Guangzhou commonly reside: Low-Rent Housing Communities, Commodity Housing Communities, Affordable Housing Communities, Resettlement Housing Communities, and Retirement Communities. Statistical analysis indicates that the Commodity Housing Community holds the largest share (25%), followed by the Affordable Housing Community (20%), the Low-Rent Housing Community (18%), the Resettlement Housing Community (15%), and the Retirement Community (14%). Collectively, these five types encompass 92% of the older residential population in Guangzhou. The remaining 8% reside in other housing types, such as flat dormitories, which were not included in the top five.

Due to considerations of research scope, this study did not incorporate the living environments of the remaining 8% demographic, which introduces a minor limitation. However, preliminary investigations revealed that older adults residing in these excluded dwellings exhibit a high degree of residential mobility, typically finding it difficult to remain in a fixed residence for 1 year or longer. Consequently, they largely do not meet the study’s inclusion criteria (residency ≥1 year).

##### 3.1.1.3 Characteristics of selected community types

The specific characteristics of the five selected community types are as follows:

(1) Low-Rent Housing Community: A government-funded, lease-only social security housing program for low-income city dwellers.
(2) Commodity Housing Community: The most common type of housing that is based on the market and gives people individual property rights and a variety of living spaces, from high-rises to villas. High prices make it hard for low-income people to get in, even though the management is better.
(3) Affordable Housing Community: These are new homes that are offered for less than the market value, which is different from low-rent housing. They go after people who cannot afford to buy goods on the market, and the costs go down thanks to government land and tax breaks.
(4) Resettlement Housing Community: Built to house people from rural or urban areas who have to move because of urbanization and infrastructure developments. These communities are usually on the edges of cities, where the government gives out state-owned apartments as compensation.
(5) Retirement Community: In this study, this type means state-funded, non-profit nursing facilities for people who need help. They offer medical and nursing care around the clock, mostly to older folks who have trouble moving about or cannot afford it and do not have relatives to help them.

##### 3.1.1.4 Spatial distribution and final sample selection

The five community types follow a generally consistent spatial pattern based on land value and function. Commodity housing is typically located in central urban areas where land value is highest. Conversely, Low-Rent and Affordable Housing communities, as government-subsidized forms, are usually situated in peri-urban areas where land prices are relatively lower. Resettlement Housing communities are generally established in outer suburban or exurban areas, closer to rural regions. Retirement communities exhibit greater locational flexibility, found in both city centers and suburbs depending on accessibility and cost.

Guangzhou comprises 11 administrative districts: Yuexiu, Haizhu, Liwan, Tianhe, Baiyun, Huangpu, Huadu, Panyu, Nansha, Conghua, and Zengcheng. Utilizing housing data from the Official Website of Guangzhou City (38), and Lianjia Guangzhou (Common websites for publishing real estate information in Chinese cities), a stratified sampling method was employed. One representative community of each of the five types was selected within each of the 11 districts, resulting in a total of 55 sample communities. This selection strategy ensured comprehensive coverage across the city center, suburban areas, outlying districts, and urban–rural fringe zones.

#### 3.1.2 Participants and sampling

To be eligible, participants had to be rural-to-urban migrants aged 60 or older who had lived in the city for more than a year (one per household). A stratified sampling approach was applied to ensure a representative sample of the older population across 5 different residential types within each of Guangzhou’s 11 districts. Employing Cochran’s (39) formula, the required sample size for this study was calculated as 384. To account for potential data loss, approximately 5% additional participants were recruited. Ultimately, responses were received from 412 participants, comprising 216 responses from an online questionnaire platform and 196 responses from face-to-face survey.

#### 3.1.3 Data collection procedure and sensitivity analysis

This study collected data through two methods: an online survey using electronic questionnaires and a face-to-face survey using paper questionnaires. From September 2023 to April 2024, data was collected from 55 typical residential communities in Guangzhou utilizing a mixed-mode technique. All surveys were conducted anonymously, with participants informed of the study’s purpose and participating voluntarily.

##### 3.1.3.1 Recruitment strategy

To reduce coverage bias, both online and offline methods were used. Online recruitment used Residents’ Committee WeChat groups and hobby groups for seniors, such square dance groups. To close the digital gap, family members helped seniors who did not have access to technology under rigorous rules to make sure they could make their own choices. This channel exhibited a relatively higher rate of non-response and ineligibility (70% non-response), likely due to the passive nature of group messaging and the strict age and migration criteria.

Offline recruitment coupled intercepts in public places with visits from community staff to people’s homes, making sure that older folks who live alone could get to them. First, targeted recruitment facilitated by community committees yielded a high cooperation rate (>90%) due to the endorsement of trusted staff. Second, to mitigate the potential “cooperativeness bias” of committee referrals, random intercepts and snowball sampling were employed. While exact non-response rates for this random component were difficult to quantify, field observations indicated that these participants provided essential diversity, capturing individuals less connected to community administration.

##### 3.1.3.2 Standards and quality control

A strict structure for standards was put in place to make sure that the results were reliable. Five bilingual Research Assistants (Master’s candidates) received extensive training in ethical compliance and impartial probing methodologies. Standardized introduction scripts and stringent controls over the setting (for example, employing semi-private spaces for offline interviews and stress-testing digital platforms) made sure that procedures were followed. There were three parts to the quality control system: a pilot study with 54 participants, random field spot checks of about 5% of offline cases, and digital attention checks.

##### 3.1.3.3 Data hygiene

Systematic data cleansing was done on 412 raw replies (216 online and 196 offline). Some of the reasons for not include someone were a lot of missing data, completion times that were too short (“speeders”), and failed attention tests. The study ultimately yielded 385 valid questionnaires, satisfying the sample size criterion and enabling progression to the data analysis phase.

##### 3.1.3.4 Sensitivity analysis

Before conducting the main analysis, a sensitivity analysis was performed to evaluate potential mode effects between online (*n* = 196) and offline (*n* = 189) participant groups.

First, Group Statistics and Independent Samples T-tests revealed no statistically significant differences between the two groups regarding sociodemographic characteristics—Age (*p* = 0.443), Education level (*p* = 0.441), Income (*p* = 0.551), Length of residence (*p* = 0.428), and Health (*p* = 0.775) —nor in the key constructs of Environmental Adaptation (*p* = 0.315) and Life Satisfaction (*p* = 0.287). Chi-square test results indicated that there were no statistically significant differences between the two groups across the other sociodemographic variables, including Gender (*p* = 0.513), Marital Status (*p* = 0.113), Religious Beliefs (*p* = 0.868), and Community Type (*p* = 0.302). Similarly, no significant variations were found for Household Structure (*p* = 0.142), Length of Residence (*p* = 0.428), and Hukou status (*p* = 0.831). The analysis revealed no statistically significant (all *p* > 0.05) differences between the two groups across any of the 11 demographic indicators and the 2 core research variables.

Second, to further test robustness, a Regression Sensitivity Analysis was conducted. We introduced “Survey Mode” as a control variable in the baseline regression model. The results indicated that Survey Mode was not a significant predictor of Life Satisfaction (beta = 0.016, *p* = 0.686), and the coefficient of the primary independent variable (Environmental Adaptation) remained stable (changing negligibly from 0.865 to 0.864). These results confirm that the recruitment approach did not introduce significant selection bias.

### 3.2 Instruments

The items included in the questionnaire were all scored on a 5-point Likert scale in which ‘1’ indicated ‘Strongly Disagree’ and ‘5’ indicated ‘Strongly Agree.’

#### 3.2.1 Life satisfaction

This study primarily used the Satisfaction With Life Scale (SWLS) developed by Diener et al. (40), which is among the most prevalent tools for assessing life satisfaction. The SWLS is a 5-point Likert scale that asks individuals to rate their agreement with statements such as ‘In most ways my life is close to my ideal.’ The reliability and validity of the SWLS have been repeatedly demonstrated, including that the Chinese version exhibits good internal consistency and construct validity (41, 42). In the current study, the reliability of SWLS for older migrants in Guangzhou was 0.929 by Cronbach’s alpha, which reached a high level of reliability. Further, the KMO (0.885) and Bartlett’s test (χ^2^ = 1658.315, df = 10, *p* < 0.001) indicate that the scale is acceptable for factor analysis.

#### 3.2.2 Perceived living environment

Drawing upon prior research concerning neighborhood living conditions (43–45), we developed a Perceived Living Environment Scale as a benchmark for assessing residential environments. To comprehensively evaluate the impact of various living environment factors on life satisfaction while aligning with China’s housing context, this scale was formulated based on the Chinese Age-Friendly Community Evaluation System proposed by Yu et al. (23). This framework comprehensively summarizes key community environmental elements through physical and social environment indicators, balancing indoor and outdoor settings while considering both intra-household and external neighborhood social interactions. It provides the foundational research basis for this study’s evaluation.

This scale comprises eight indicators reflecting the perceived living environment, including five indicators [neighborhood environment (NE), environmental performance (EP), housing environment (HE), services and facilities (SF), road and traffic (RT)] encompassing 22 items, and three indicators [social engagement (SE), social inclusion (SI), communication and information (CI)] comprising 11 items. The questionnaire survey in this part asks participants to rate their agreement with statements such as ‘There are many public places to go within easy walking distance of my home.’ Good internal consistency of the ‘perceived living environment’ scale was evidenced by a Cronbach’s alpha coefficient of 0.878. The suitability for factor analysis was supported by a KMO value of 0.838 and a significant Bartlett’s test of sphericity (χ^2^ = 6387.914, df = 528, *p* < 0.001).

#### 3.2.3 Environmental adaptation

The conceptual framework integrates six specific theoretical perspectives to operationalize “Environmental Adaptation” as a holistic survival and adjustment mechanism for older migrants:

(1) Physiological Adaptation (Based on Evolutionary & Ecological Theory): Drawing from Darwin (46) and Schluter (47), this dimension captures the organism’s homeostatic maintenance in response to physical environmental shifts. For older migrants, whose physiological resilience declines with age (48), this involves physical acclimatization to the new urban climate and living conditions.
(2) Behavioral Adaptation (Based on Human Behavioral Ecology): Grounded in behavioral plasticity and niche construction (49), this refers to the active modification of daily habits (e.g., diet, mobility) to optimize survival in the new urban “niche.”
(3) Social Adaptation (Based on Ecological Systems Theory): Distinct from general social interaction, this dimension is defined by Bronfenbrenner’s (50) Microsystem and Exosystem. It measures how migrants rebuild their ecological networks (family, neighbors, community services) in the absence of their original rural support systems (51).
(4) Psychological Adaptation (Based on Stress Adaptation Theory): Specifically grounded in Lazarus and Folkman’s (52) Cognitive Appraisal Model. It refers to the cognitive process of evaluating stressors (primary appraisal) and assessing coping resources (secondary appraisal). It is strictly defined as the mental restructuring of one’s identity and goals in the new environment (53).
(5) Resilience/Emotional Adaptation (Based on Resilience Theory): distinct from cognitive appraisal, this dimension draws on Masten (54) to represent the affective capacity to “bounce back” (homeostasis recovery). It captures the emotional regulation capability required to withstand the shock of displacement, which is critical for successful aging (55).

A review panel comprising six experts—including gerontologists and urban scholars—examined the initial project portfolio to ensure its relevance and clarity. Furthermore, through factor analysis of indicators using a sample of 54 pilot studies, this research identified eight primary dimensions of environmental adaptation variables. Perceived environment discrepancy (EA1), perceived person discrepancy (EA2), psychological adaptation (EA3), emotional adaptation (EA4), physiological adaptation (EA5), behavioral adaptation (EA6), social adaptation (EA7), and cultural adaptation (EA8) were finally identified to assess the mediating variable of ‘environmental adaptation’ in this study. The ‘environmental adaptation ‘scale demonstrated good internal consistency, with a Cronbach’s alpha of 0.840. Furthermore, the data’s suitability for factor analysis was confirmed by a Kaiser-Meyer-Olkin (KMO) measure of 0.864 and a significant Bartlett’s test of sphericity (χ^2^ = 1001.162, df = 28, *p* < 0.001).

#### 3.2.4 Factor analysis

Exploratory factor analysis (EFA) revealed that, following orthogonal rotation, 10 common factors with eigenvalues exceeding one were derived from the 46 measurement items in the questionnaire data, cumulatively explaining 72.147% of the variance. The factor loadings for all items exceeded 0.60. This indicates that these 10 factors account for 72.147% of the information from the 46 items, suggesting that the extracted factors effectively clarify the information contained within the overall questionnaire data. These 10 identified factors represent neighborhood environment (NE), environmental performance (EP), housing environment (HE), services and facilities (SF), road and traffic (RT), social engagement (SE), social inclusion (SI), communication and information (CI), environmental adaptation (EA), and life satisfaction (LS).

Confirmatory factor analysis (CFA) based on the aforementioned 10 factors indicates that the factor structure exhibits acceptable goodness of fit. A measurement model is considered perfect fit if the chi-square value is zero while model fit is obtained from a not significant chi-square. However, due to the sensitivity of chi-square to the sample size, statisticians suggest that researchers to use multiple indices to evaluate the model fit (56). Results in Table 1 revealed that the 10-factor measurement model was correctly adjusted to the data, i.e., Chi-square χ^2^(df = 944, *p* = 0.000) = 1914.434, the degree of freedom (χ^2^/df) = 2.028, Comparative fit index (CFI) = 0.901, Tucker-Lewis index (TLI) = 0.912 and the root-mean-square error of approximation (RMSEA) = 0.052.

**Table 1:** Measurement model fitting parameters.

Statistical test	χ^2^/df	TLI	CFI	RMSEA
Adaptation standard	<3	>0.9	>0.9	<0.10
Value	2.028	0.912	0.901	0.052

χ^2^ = 1914.434, df = 944, *p* = 0.000.

The CFA analysis results demonstrate that the standardized factor loadings for the indicators of the 10 latent variables in the measurement model exceed 0.5, with all factor coefficients being highly significant (*p* = 0.000). This indicates a robust explanatory capacity of the measurement model. Furthermore, the combined reliability (CR) of the 10 latent variables surpasses 0.7, signifying strong internal consistency and convergent validity of the measurement model.

The discriminant validity of a measurement model pertains to the extent to which the attributes of the created latent variables exhibit low correlation or notable distinctions from other latent variables (63). The assessment of discriminant validity necessitates that the measurement model successfully meets both the combined reliability and convergent validity criteria, and that the square root of the Average Variance Extracted (AVE) for any latent variable exceeds the absolute value of the correlation coefficient between those variable and other latent variables. Only in this manner can it be asserted that the discriminant validity among the latent variables is satisfactory.

The discriminant validity test results of this study are shown in Table 2, where the square root values of the AVE for all 10 factors in the model are greater than the maximum of the absolute values of the correlation coefficients between the factors, which indicates that the model possesses strong discriminant validity, meaning the characteristics of each latent variable are markedly distinct from those of other latent variables.

**Table 2:** Discriminant validity: correlation coefficient with AVE square root value.

Factor (Latent variable)	(NE)	(EP)	(SF)	(RT)	(HE)	(SE)	(SI)	(CI)	(EA)	(LS)
Neighborhood environment (NE)	**0.667**									
Environmental performance (EP)	0.276	**0.769**								
Services and facilities (SF)	0.439	0.322	**0.731**							
Road and traffic (RT)	0.365	0.197	0.665	**0.678**						
Housing environment (HE)	0.363	0.136	0.528	0.464	**0.649**					
Social engagement (SE)	0.269	0.218	0.286	0.308	0.176	**0.642**				
Social inclusion (SI)	0.391	0.106	0.414	0.413	0.454	0.186	**0.768**			
Communication and information (CI)	0.115	0.089	0.115	0.063	0.017	−0.002	0.049	**0.835**		
Environmental adaptation (EA)	0.244	0.253	0.318	0.202	0.157	0.251	0.168	0.093	**0.633**	
Life satisfaction (LS)	0.064	0.133	0.159	0.132	0.111	0.072	0.090	0.066	0.106	**0.857**

Bolded diagonal figures are the square root of AVE, the remaining figures denote correlation coefficients.

In conclusion, the results of the factor analysis indicated that the measurement model in this study exhibited a good fit, along with strong reliability and validity.

### 3.3 Statistical analysis

The Software Package for Social Science (SPSS) version 26.0, a widely used software for statistical analysis in social science research, was utilized for data analyses such as factor analysis, description data, variance, regressions, and mediating analyses in the questionnaire survey’s data transformation process. Firstly, descriptive statistics were employed to characterize participants’ sociodemographic attributes. Secondly, the t-test and analysis of variance (ANOVA) were conducted to explore and characterize (rather than just test for significance) the features presented by the level of life satisfaction and the level of environmental adaptation of older migrants with varying sociodemographic profiles. Finally, environmental adaptation was examined as a potential mediator between perceived living environment and life satisfaction. The analysis employed the causal steps approach by Baron and Kenny (57) alongside the bootstrapping method (58), while accounting for the influence of sociodemographic characteristics. Guided analysis tests (with the sampling process repeated 1,000 times) were employed to examine the model’s overall, indirect, and direct effects. An indirect effect (the mediating effect in the study) was deemed statistically significant if the 95% confidence interval (CI) excluded zero.

### 3.4 Ethics statement

A letter of approval from the University of Malaya Research Ethics Committee (UMREC) was obtained for this study before any formal surveys and interviews were conducted. The Reference Number of the approval letter is UM.TNC2/UMREC_3109.

## 4 Results

### 4.1 Descriptive and variance analysis findings

Table 3 shows the sociodemographic characteristics of older migrants in Guangzhou. In this study, 54.8% of respondents were male, while 45.2% were female. The largest age cohort among respondents (51.9%) was aged 65 to 79, whereas those aged 90 and above constituted only 6.5%. Data on educational attainment indicates that 20.3% of respondents had never received formal schooling, while only 6.2% held a bachelor’s degree or higher qualification. Over 60% of respondents were married, and more than 85% reported a monthly income exceeding ¥3,000 (about $422). Table 4 summarizes the distribution of the 385 survey respondents in terms of *hukou*, housing structure, community type, and length of residence, which are demographic characteristics related to residential status. Among the respondents, 61.6% have relocated their *hukou* to urban regions, indicating a tendency of urban migration within this older demographic. The household structure data indicate that 4.9% of respondents reside independently, and the remaining individuals inhabit multi-generational homes. A substantial majority (63.4%) of participants inhabit resettlement housing communities, maybe indicative of urban migration policy or economic limitations. A significant proportion of respondents (58.4%) have resided in their current dwelling for 5–10 years, reflecting a degree of stability in housing.

**Table 3:** Sociodemographic characteristics and variance analysis of LS and EA among older migrants.

Variable	Category	N (%)	Life satisfaction (LS)		Environmental adaptation (EA)	
			t/F	*p*	t/F	*p*
Gender	1 = male, 2 = female.	211(54.8) 174(45.2)	0.667	0.415	0.701	0.403
Age	1 = 60–65, 2 = 66–79, 3 = 80–89, 4 = 90 & above.	110(28.6) 200(51.9) 50(13.0) 25(6.5)	0.492	0.688	2.276	0.079
Education level	1 = Never went to, 2 = Primary, 3 = Junior high, 4 = High, 5 = Technical school, 6 = Bachelor & higher.	78(20.3) 55(14.3) 58(15.1) 74(19.2) 96(24.9) 24(6.2)	0.533	0.751	3.415	0.005
Marital status	1 = married, 2 = others (widowed, divorced, separated or never married)	235(61.0) 150(39.0)	3.236	0.073	0.001	0.978
Religious beliefs	1 = Buddhism, 2 = Christianity, 3 = Islam, 4 = Others, 5 = None.	63(16.4) 114(29.6) 46(11.9) 45(11.7) 117(30.4)	2.196	0.069	1.022	0.396
Income	1 = Below¥ 1,000, 2 = ¥1,000-3,000, 3 = ¥3,000–5,000, 4 = ¥5,000 & above.	13(3.4) 34(8.8) 184(47.8) 154(40.0)	1.207	0.307	3.451	0.017
Health status	1 = Poor, 2 = Fair, 3 = Good, 4 = Very Good, 5 = Excellent	68(17.7) 78(20.3) 93(24.2) 68(17.7) 78(20.3)	0.846	0.497	0.533	0.712

**Table 4:** Residential status and variance analysis of LS and EA among older migrants.

Variable	Category	N (%)	Life satisfaction (LS)		Environmental adaptation (EA)	
			t/F	*p*	t/F	*p*
*Hukou*	1 = Transferred to the urban area, 2 = Remains in the rural area.	237(61.6) 148(38.4)	0.040	0.842	0.151	0.698
Household structure	1 = Living with spouse, 2 = Living with adult children and grandchildren, 3 = Living with spouse and adult children, 4 = Living with spouse, adult children and grandchildren, 5 = Living alone.	59(15.3) 207(53.8) 48(12.5) 52(13.5) 19(4.9)	0.802	0.525	0.669	0.613
Community types	1 = Low-rent housing, 2 = Commodity housing, 3 = Affordable housing, 4 = Resettlement housing, 5 = Retirement community.	21(5.5) 51(13.2) 63(16.4) 246(63.9) 4(1.0)	0.196	0.940	1.060	0.376
Length of residence	1 = About 1 year, 2 = 1-3 years, 3 = 4-5 years, 4 = 6-10 years, 5 = 10 years above.	15(3.9) 14(3.6) 68(17.7) 225(58.4) 63(16.4)	1.109	0.352	0.269	0.898

The results of t-test and the ANOVA of life satisfaction level across 11 different groupings among older migrants in Tables 3, 4 show that, from all the final *p*-values which are all above 0.05, the level of life satisfaction shows no significant differences. Although variations in sociometric characteristics do not substantially influence overall life satisfaction levels, Weiss (59) demonstrates that a heightened F-ratio (*F* > 1) suggests that the observed differences across group mean values are unlikely to have occurred by random chance. The *F*-value in ‘marital status’, ‘religious beliefs’, ‘income’ and ‘length of residence’ groups are higher than 1.0, the comparisons of mean values of different options presented in these 4 groups reflect nuanced differences in levels of life satisfaction.

Tables 3, 4 also present the results of t-test and the ANOVA of the level of environmental adaptation across 11 different groupings, and from all the final p-values presented in the tables, it is clear that the level of environmental adaptation exhibits significant differences in two dimensions, namely, level of education and income, which indicates that differences between levels of education, and differences between levels of income significantly affect the level of environmental adaptation.

Given the assumption of equal variances (Income: *p* = 0.311/ Education Level: *p* = 0.928), the LSD technique is frequently employed as a *post hoc* test to identify homogenous subsets and facilitate pairwise comparisons. The group of ‘Education Level’ have 6 options, the results indicate a substantial disparity in environmental adaptation between option 3 (Junior high school) and options 1, 2, 4, 5, and 6 (the remaining five educational levels). Furthermore, the mean comparisons of the six options suggest that older migrants with a junior high school education exhibit a markedly higher average level of environmental adaptation than their counterparts with other educational qualifications (consistent with those with lower and higher level education). The ‘Income’ group comprises four alternatives, with results revealing a large differential in environmental adaptation between option 2 (¥1,000-3,000) and option 3 (¥3,000-5,000), as well as a notable difference between option 2 and option 4 (¥5,000 and above). Moreover, the mean comparisons of the four alternatives indicate that older migrants with an income range of ¥1,000–3,000 demonstrate a superior average level of environmental adaptability compared to those in the other two income brackets.

### 4.2 The mediating analysis of environmental adaptation

The analysis of mediating effects is to test whether the effect of the 8 dimensions of ‘Perceived Living Environment ‘on the ‘Life Satisfaction’ is partially or fully explained by the ‘Environmental Adaptation’ of the older migrants in Guangzhou, even when main sociodemographic factors are controlled for. Not only that, mediated effects analyses seek to understand the underlying mechanisms or processes that explain how the ‘Perceived Living Environment’ influences the ‘Life Satisfaction’.

The analysis of the mediation effect is done through a three-step regression analysis, (1) the impact of ‘Perceived Living Environment’ on Life Satisfaction (LS), (2) the impact of ‘Perceived Living Environment’ on Environmental Adaptation (EA), (3) The impact of ‘Perceived Living Environment’ and ‘Environmental Adaptation (EA)’ on ‘Life Satisfaction (LS)’. Among them, whether there is a mediating effect mainly depends on the results of the third step regression analysis.

The ‘Perceived Living Environment’ variable has 8-dimension variables (5 Physical aspect and 3 social aspect), so there are 8 mediation effect models in this section that are tested in the above way.

Table 5 presents the mediating role of environmental adaptation in the relationship between five physical living environment factors and life satisfaction. According to the mediation analysis in Model 1, the regression coefficient for environmental adaptation in the third step is 0.210, with a *p*-value below 0.05, indicating a significant mediating effect of environmental adaptation between neighborhood environment and life satisfaction. This study then used the Bootstrap method with 1,000 samples to evaluate the robustness of the aforementioned mediating effects. The primary emphasis is on the 95% confidence interval of a*b, serving as an indicator of the mediating impact. If the interval excludes 0, it indicates that the mediation’s robustness is confirmed. Following further validation via the Bootstrap method (Table 6), the Model 1 was confirmed, with results indicating that environmental adaptability fully mediated the relationship between neighborhood environment (NE) and life satisfaction.

**Table 5:** Mediation effects test: physical living environment factors (5)—environmental adaptation—life satisfaction.

Factors of perceived living environment	Item	Life satisfaction (LS)	Environmental adaptation (EA)	Life satisfaction (LS)
Model 1: Neighborhood environment (NE)	NE	0.158(1.296)	0.286**(4.843)	0.098(0.783)
	EA			0.210*(1.969)
	*R* ^2^	0.037	0.094	0.047
	Adjust R^2^	0.006	0.065	0.014
Model 2: Environmental performance (EP)	EP	0.266**(2.734)	0.238**(5.034)	0.225*(2.246)
	EA			0.170(1.600)
	*R* ^2^	0.052	0.098	0.058
	Adjust R^2^	0.021	0.069	0.025
Model 3: Housing environment (HE)	HE	0.259*(2.207)	0.164**(2.820)	0.226*(1.912)
	EA			0.202(1.936)
	*R* ^2^	0.045	0.057	0.055
	Adjust R^2^	0.015	0.026	0.022
Model 4: Services and facilities (SF)	SF	0.352***(3.228)	0.323***(6.157)	0.306***(2.675)
	EA			0.143(1.326)
	*R* ^2^	0.059	0.126	0.064
	Adjust R^2^	0.029	0.098	0.031
Model 5: Road and traffic (RT)	RT	0.263**(2.751)	0.179**(3.781)	0.230*(2.371)
	EA			0.183(1.743)
	*R* ^2^	0.052	0.072	0.060
	Adjust R^2^	0.022	0.042	0.027

*p < 0.05, **p < 0.01.

**Table 6:** Validation of mediation model 1.

Neighborhood environment (NE)→Environmental adaptation (EA)→Life satisfaction (LS)									
Item	Symbol	Effects	Effect value	95% CI		Std. error SE value	t-value	*p*-value	Conclusion
				Lower limit	Upper limit				
NE→EA→LS	a*b	Indirect effects	0.060	0.002	0.061	0.016	3.728	0.000	Full mediating effect
NE→EA	a	X→M	0.286	0.170	0.402	0.059	4.843	0.000	
EA→LF	b	M→Y	0.210	0.001	0.419	0.107	1.969	0.050	
NE→LF	c’	Direct effects	0.098	−0.147	0.343	0.125	0.783	0.434	
NE→LF	c	Total effect	0.158	−0.081	0.397	0.122	1.296	0.196	

In Model 2, the coefficient for environmental adaptation is 0.170, with a p-value greater than 0.05, suggesting that the mediating effect is not statistically significant in this case. Model 3 results show that environmental adaptation do not have a significant mediating role between housing environment and life satisfaction (coefficient = 0.202, *p* > 0.05). For Model 4, the coefficient of environmental adaptation is 0.143, yet the p-value exceeds 0.05, indicating the absence of a significant mediation effect. Finally, Model 5 demonstrates that environmental adaptation does not significantly mediates the association between road and traffic conditions and life satisfaction, with a coefficient of 0.183 and a p-value greater than 0.05 in the third-step regression.

Table 7 presents the mediating effect of environmental adaptation on life satisfaction across three social residential environment factors. In Model 6, the third-step regression results indicate that environmental adaptation does not function as an mediate role between social engagement and life satisfaction (coefficient = 0.203, *p* > 0.05). Similarly, Model 7 shows no significant mediating effect of environmental adaptation between social inclusion and life satisfaction, with a coefficient of 0.200 (*p* > 0.05).

**Table 7:** Mediation effects test: social living environment factors (5)—environmental adaptation—life satisfaction.

Factors of perceived living environment	Item	Life satisfaction (LS)	Environmental adaptation (EA)	Life satisfaction (LS)
Model 6: Social engagement (SE)	SE	0.177(1.582)	0.252**(4.628)	0.126(1.098)
	EA			0.203(1.910)
	*R* ^2^	0.039	0.089	0.049
	Adjust R ^2^	0.008	0.060	0.015
Model 7: Social inclusion (SI)	SI	0.163*(2.093)	0.122**(3.184)	0.138(1.760)
	EA			0.200(1.917)
	*R* ^2^	0.044	0.062	0.054
	Adjust R ^2^	0.013	0.032	0.020
Model 8: Communication and information (CI)	0.064(1.135)	0.047*(1.657)	0.054(0.954)	0.064(1.135)
				0.222*(2.138)
	0.036	0.044	0.048	0.036
	0.005	0.013	0.015	0.005

*p < 0.05, **p < 0.01.

Furthermore, Model 8 demonstrates that environmental adaptation serves as a significant mediator between communication and information (CI) and life satisfaction, as evidenced by a coefficient of 0.222 (*p* < 0.05). However, as we subsequently reported in Table 8, this effect was found to be non-robust. Therefore, the study ultimately concluded that environmental adaptation does not mediate the relationship between communication and information (CI) and life satisfaction.

**Table 8:** Validation of mediation model 8.

Communication and information (CI)→Environmental adaptation (EA)→Life satisfaction (LS)									
Item	Symbol	Effects	Effect value	95% CI		Std. error SE value	t-value	*p*-value	Conclusion
				Lower limit	Upper limit				
CI→EA→LS	a*b	Indirect effects	0.010	−0.002	0.023	0.006	1.711	0.087	The mediating effect was not significant
CI→EA	a	X→M	0.047	−0.009	0.102	0.028	1.657	0.098	
EA→LS	b	M→Y	0.222	0.018	0.425	0.104	2.138	0.033	
CI→LS	c’	Direct effects	0.054	−0.057	0.165	0.057	0.954	0.341	
CI→LS	c	Total effect	0.064	−0.047	0.175	0.057	1.135	0.257	

**Figure 2:**
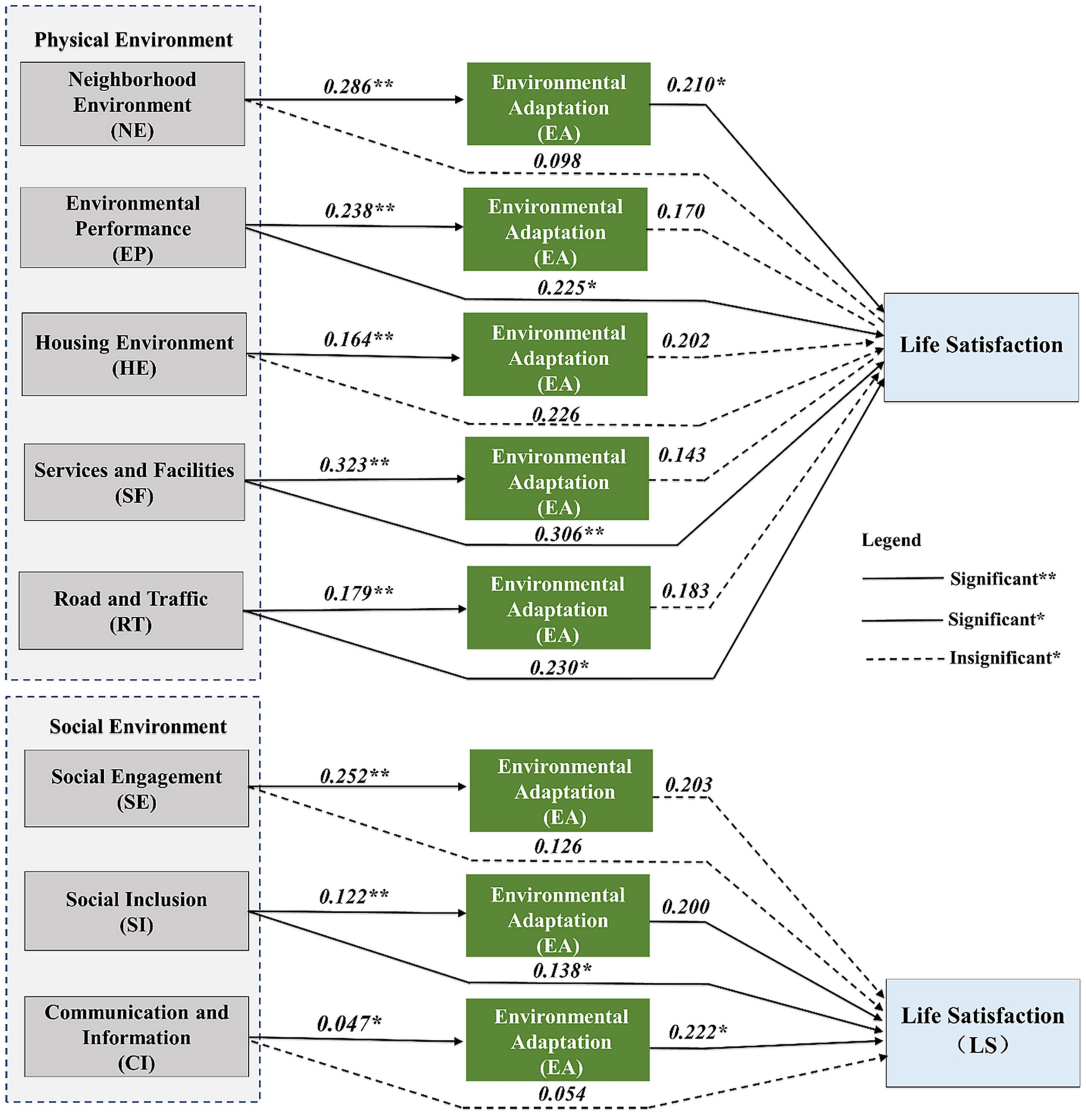
Pathways of impact of the perceived living environment on life satisfaction through environmental adaptation.

Based on the synthesis of the aforementioned mediating analyses, this study has mapped out the potential pathways of impact of the living environment factors on life satisfaction through environmental adaptation (Figure 2). This pathway may constitute the fundamental mechanism through which both the living environment and environmental adaptation predict life satisfaction of older migrants in Guangzhou.

## 5 Conclusion and discussion

Both research objectives of this study were successfully accomplished. First, the features and levels of life satisfaction and environmental adaptability of older migrants with different sociodemographic characteristics in Guangzhou, China have been identified.

(1) The ANOVA test results indicate that there are no significant differences in life satisfaction among 11 groups of sociodemographic characteristics.
(2) The environmental adaptability of older migrants is significantly impacted by differences in educational attainment and income levels. Meanwhile, differences in length of residence also produce subtle differences in older migrants’ ability to adapt to their environments.

Second, this study examined the direct and indirect effects of perceived living environment on life satisfaction, as well as the potential mediating role of environmental adaptation.

(1) Multiple regression analyses indicate that the ‘environmental performance’, ‘services and facilities’, ‘road and traffic’, and ‘social inclusion’ exhibit significantly impact on life satisfaction of older migrants. However, ‘neighborhood environment’, ‘housing environment’, ‘social engagement’ and ‘communication and information’ are factors of perceived living environment that do not have a direct impact on the life satisfaction of older migrants.
(2) The mediating analysis identifying environmental adaptation as a full mediator in the relationships of ‘neighborhood environment’ with life satisfaction.
(3) The ‘communication and information’ and ‘life satisfaction’ are neither directly nor indirectly related in this study.

### 5.1 Late-life migration in Guangzhou

The dominance of resettlement housing residents (63.9%) in our sample reflects the demographic reality of Guangzhou’s rapid urbanization, highlighting two distinct patterns of internal late-life migration in China: voluntary and involuntary. Voluntary migration is primarily driven by the ‘Older Migrants Following Children’ phenomenon (15). Under the ‘One-Child Policy’ legacy, rural parents relocate to urban centers to provide grandchild care and seek emotional support. Additionally, policies such as ‘Household Registration for Older Parents Joining Children’ in Guangzhou encourage financially capable seniors to migrate for superior urban healthcare and social services (16). Involuntary migration, however, constitutes a significant majority in the urban–rural fringe, as evidenced by our data. This pattern is driven by urban renewal and land expropriation. As Guangzhou expands, rural lands are appropriated for infrastructure, compelling residents to relocate to government-provided high-rise resettlement communities. Unlike voluntary migrants, these ‘land-lost’ older individuals face a passive transition. Our study’s focus on this specific subgroup offers a unique contribution, as their adaptation challenges are often more severe than those of voluntary migrants described in Western literature.”

The finding that environmental adaptation fully mediates the relationship between ‘neighborhood environment’ and life satisfaction is uniquely suited to the Chinese urban residential context. Unlike the open-street neighborhoods common in Western cities or the ‘acquaintance society’ of rural Chinese villages—where neighbors are familiar and spaces are accessible—modern Chinese urban housing largely consists of Gated Communities. These are enclosed, high-density vertical precincts accessed only via security checkpoints. For older migrants accustomed to the freedom of rural courtyards and close-knit social bonds, moving into these ‘concrete forests’ represents a radical spatial disruption. The physical quality of the neighborhood (e.g., green ratios or facility conditions) does not directly translate to satisfaction because the spatial form itself is alienating. Therefore, the ‘full mediation’ effect reveals a critical mechanism: the physical environment can only enhance well-being if the older migrant first successfully navigates the psychological process of ‘breaking the wall’—adapting to the loss of open space and the transition from a society of acquaintances to a society of strangers. This validates our framework as specifically responsive to the socio-spatial transformation of China’s urbanization.”

The result that there is not a significant difference in life satisfaction across the majority of sociodemographic groups (such as age, gender, or education level) shows that there is some degree of consistency in the way that older migrants view their own well-being. This suggests that the elements that have a greater impact on life happiness are universal in nature, such as the difficulties associated with migrating or the experiences that are shared by society and culture, rather than the sociodemographic features of various individuals. When it comes to policymakers, this suggests that policies that target the quality of life holistically may be just as effective as those that are tailored to specific needs.

In terms of environmental adaptability, higher education levels may correlate with greater adaptability among older migrants due to the development of advanced problem-solving skills, enhanced critical thinking abilities, and a greater capacity to process and utilize information about available resources in urban settings. Educated individuals are often better equipped to navigate complex systems, such as healthcare, transportation, and social services, which are critical for successful integration into new environments (60, 61). Furthermore, higher education is frequently associated with broader social networks and a greater sense of self-efficacy, both of which contribute to improved adaptation outcomes.

### 5.2 Development of the environmental adaptation framework

**Figure 3:**
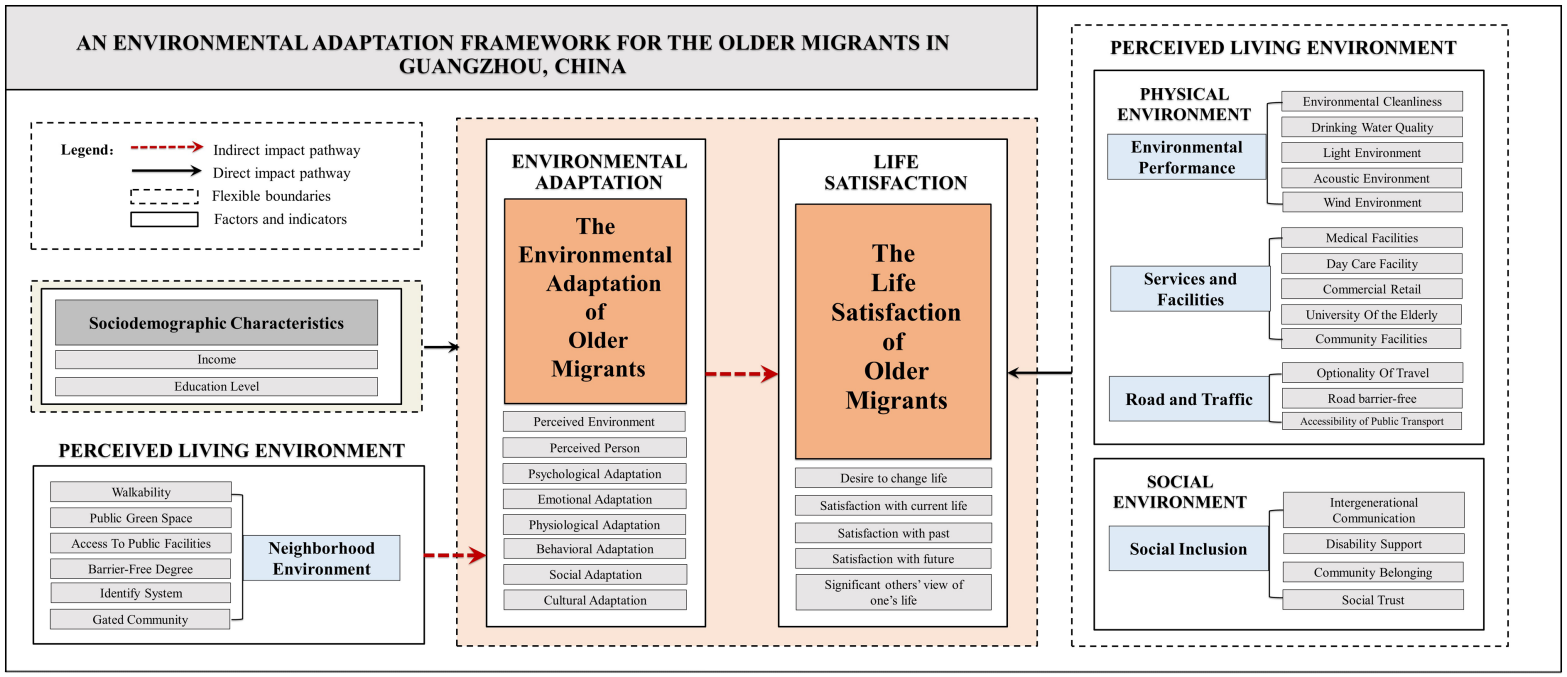
The environmental adaption framework for the older migrants in Guangzhou, China.

As illustrated in Figure 3, the research findings are synthesized into a graphical framework. This framework represents the principal outcome of the study—The Environmental Adaptation Framework for Older Migrants in Guangzhou, China—and encapsulates the core theoretical contribution of the research. This environmental adaptation framework presents the impact pathways of the living environment on life satisfaction among older migrants in Guangzhou, China. Impact pathways represent the transmission of influence through direct and indirect causal chains between variables, emphasizing their relative weight in the process (62).

In this study, the findings concluded the direct impact pathway of the sociodemographic characteristic factors of older migrants in Guangzhou as well as the factors of the perceived living environment have an impact on life satisfaction and environmental adaptation; and also found the indirect pathway of the perceived living environment’s impact on life satisfaction under the role of environmental adaptation.

Structurally, the dotted box at the top of this framework summarizes the sociodemographic characteristics that have a significant impact on levels of life satisfaction and environmental adaptation. The framework’s rightward module operationalizes four living environment determinants (environmental performance, services and facilities, road and traffic, and social inclusion), each demonstrating direct pathways on the life satisfaction of older migrant populations using black solid line arrow.

The framework’s leftward module formalizes only one living environment determinants whose effects on migrant elders’ life satisfaction are fully mediated by environmental adaptation processes - a relationship represented through dashed red vector notation, while simultaneously theorizing adaptation’s central mediating function in the well-being ecology. Environmental adaptation emerges as the core process through which physical (neighborhood environment) factors influence life satisfaction. These findings establish environmental adaptation as a necessary condition for translating improvements in neighborhood environment into enhanced life satisfaction among Guangzhou’s older migrant population.

### 5.3 Theoretical contributions and practical implications

This study advances the existing literature on environmental gerontology and migration adaptation in three critical ways. First, it extends the application of the Person-Environment (P-E) Fit model from its traditional organizational context to the domain of urban residential aging. Unlike classic P-E Fit models which focus on general environments (No distinction is made between work environments and living environments) and general (No age group specified) populations, this study operationalizes “Perceived Living Environment” and “Environmental Adaptation” specifically for the rural-to-urban older migrant population. By identifying the specific living environmental dimensions relevant to this group, the framework addresses the unique “dual adaptation” challenge (physical and social) faced by older migrants in rapidly densifying cities.

Second, the study empirically refines the “Age-Friendly City” indicator system for migrant populations. The evaluation system introduced by Yu et al. (23) lacks empirical validation for the measurement of age-friendly communities for Chinese older people and older migrants, particularly based on empirical surveys assessing their satisfaction with usage. While previous models often treat environmental factors as a monolith, our multiple regression analyses reveal distinct associate patterns. This distinction advances current knowledge by highlighting that not all “friendly” environmental features directly translate to satisfaction for migrants; some require active adaptation processes to become beneficial.

Third, and most importantly, the framework elucidates the specific *mechanisms* of adaptation. The study moves beyond treating adaptation as a generic black box by identifying its mediating role. A key theoretical contribution is the finding that environmental adaptation functions as a full mediator in the relationship between ‘neighborhood environment’ and life satisfaction. This suggests that for older migrants, improvements in the physical neighborhood (e.g., green spaces, safety) do not inherently relate to well-being improving unless they facilitate the psychological and behavioral process of adaptation. Furthermore, we found that ‘communication and information’ are neither directly nor indirectly related to life satisfaction in this specific context, suggesting that digital inclusion frameworks may need re-evaluation for this specific demographic.

By mapping these specific direct and indirect pathways, the proposed framework transforms the P-E Fit concept into a testable, operational model for understanding the complex well-being ecology of aging migrants.

The proposed Environmental Adaptation Framework offers actionable insights for urban policymakers and planners. To bridge the gap between theoretical modeling and practical application, this study proposes a three-tiered strategy to mobilize the Environmental Adaptation Framework for urban governance in Guangzhou and beyond.

First, the Framework as a Diagnostic Audit Tool: Urban planners and community managers can utilize the verified variables of our framework as a ‘Diagnostic Matrix’ for assessing the age-friendliness of residential communities. Instead of generic evaluations, planners can systematically score communities on ‘Environmental Performance,’ ‘Service Accessibility,’ and ‘Social Inclusion.’ For instance, in resettlement communities where we found ‘Neighborhood Environment’ relies on adaptation to influence satisfaction, the framework directs planners to identify physical barriers (e.g., lack of communal seating, poor wayfinding) that impede this adaptation process.

Second, informing ‘Micro-Regeneration’ policy. The findings provide empirical evidence to guide Guangzhou’s ongoing ‘Micro-regeneration’ policy. Currently, renovation efforts often focus on hard infrastructure (e.g., facade painting). Our framework argues for a shift toward ‘Adaptive Infrastructure.’ In terms of physical design, we recommend mandating the inclusion of ‘Therapeutic Landscapes’ (e.g., sensory gardens, sheltered walking loops) in gated communities to alleviate the stress of high-density living. For social policy, recognizing the critical role of ‘Social Adaptation’ is important. We advocate for funding community-based organizations (CBOs) to run ‘Bridge-Building’ programs. These programs would specifically target involuntary migrants in resettlement housing, facilitating their integration into the local social fabric through dialect-inclusive activities and peer-support networks.

Third, refinement via participatory co-design. To ensure the framework remains relevant, future implementation will adopt a participatory action research approach. We propose organizing ‘Co-design Workshops’ where older migrants are not just subjects but active partners. By involving them in the evaluation of their living environments using our framework, we can refine the indicators to better capture their evolving needs. This bottom-up approach ensures that urban interventions are not merely top-down impositions but are co-created solutions that genuinely enhance the environmental adaptability of the aging population.

### 5.4 Limitations and future research

Although this study offers theoretical innovations regarding the adaptation of older migrants in China, several limitations should be acknowledged, which outline critical directions for future inquiry. First, regarding the research design, the study employed cross-sectional data covering environmental adaptation among older migrants approximately 1–10 years post-relocation. Adaptation is a dynamic process; the mediating role of environmental adaptation may shift from a “survival mechanism” to a “thriving mechanism” as residence duration increases. Future studies should employ longitudinal surveys to track cohorts over time, broadening the scope of results to understand how the specific pathways between the living environment and life satisfaction evolve at different stages of migration. Second, regarding generalizability, the sample was context-specific to Guangzhou, a high-density Tier-1 city in Southern China. Given China’s vast territory, these findings may not be fully generalizable to regions with distinct geographical, climatic, and cultural contexts. To validate and expand the proposed Environmental Adaptation Framework, future research should conduct multi-scalar comparative analyses across contrasting urban typologies:

(1) Climatic and Cultural Contrasts: Comparing Guangzhou with Northern cities (e.g., Harbin or Shenyang) to examine how extreme climate differences influence physiological adaptation strategies.
(2) Urban Hierarchy: Extending the study to small counties or towns to investigate “in-situ urbanization,” where rural elders move to nearby towns—a pattern distinct from long-distance metropolitan migration.
(3) Migration Types: Comparing economic migrants in Tier-1 cities (e.g., Beijing, Shenzhen) with seasonal “snowbird” migrants in amenity-led destinations (e.g., Sanya, Xiamen), where environmental performance might outweigh social integration as a predictor of satisfaction.

Despite these limitations, this study represents an initial endeavor to understand how and why Chinese older migrants’ perceptions of their community living environment—primarily the physical and social conditions of their dwelling—correlate with their life satisfaction. The findings provide fresh evidence for the positive role of community environments in enhancing the quality of life for specific older populations, revealing that environmental adaptation underpins this positive association. Furthermore, the study develops a detailed framework to clarify the mechanisms by which the living environment affects the life satisfaction of older migrants. This framework offers theoretical insights into the relationships among housing, urban infrastructure, social integration, and well-being, while also functioning as a practical guide for policymakers and urban planners in creating age-inclusive environments that improve the quality of life for older migrants.

## Data Availability

The original contributions presented in the study are included in the article/Supplementary material, further inquiries can be directed to the corresponding author.

## Footnotes

1 One-child policy was introduced in 1979 and concluded in 2016 following the full implementation of the two-child policy. It aimed to control population size and alleviate the social, economic and environmental challenges facing China.

2 The official website: https://zfcj.gz.gov.cn/
