# Supplementary material for: Developing an environmental adaptation framework for older migrants in Guangzhou, China
Source: Front Public Health. 2026 Apr 9;14:1756061. doi: 10.3389/fpubh.2026.1756061 (PMC13102619; doi:10.3389/fpubh.2026.1756061)
Supplement: Supplementary file 1 [file Table_1.DOCX]

Supplementary Material

# Supplementary Tables

**Supplementary Table 1.** Sensitivity analysis: Comparison of the sociodemographic characteristics and core research variables between the online and offline samples.

| **Variable** | **(Online Mean)** | **(Offline Mean)** | **t** | **P (Sig.)** | **Result** |
| --- | --- | --- | --- | --- | --- |
| **Age** | 1.99 | 2.05 | -0.767 | **0.443** | No |
| **Education Level** | 3.51 | 3.42 | 0.771 | **0.441** | No |
| **Income** | 2.59 | 2.53 | 0.596 | **0.551** | No |
| **Length of Residence** | 3.55 | 3.65 | -0.793 | **0.428** | No |
| **Health** | 3.88 | 3.9 | -0.286 | **0.775** | No |
| **Environmental Adaptation** | 3.86 | 3.82 | 1.006 | **0.315** | No |
| **Life Satisfaction** | 3.91 | 3.86 | 1.066 | **0.287** | No |
| Note: No significant differences were found between groups (p > 0.05). | | | | | |

**Supplementary Table 2.** Sensitivity analysis: Comparison of the other sociodemographic characteristics between the online and offline samples.

| **(Variable)** | **Chi-Square (χ2)** | **df** | **P (Sig.)** | **Result** |
| --- | --- | --- | --- | --- |
| **Gender** | 0.428 | 1 | **0.513** | No |
| **Marital Status** | 2.536 | 1 | **0.111** | No |
| **Religious Beliefs** | 1.258 | 4 | **0.868** | No |
| ***Hukou*** | 0.046 | 1 | **0.831** | No |
| **Household Structure** | 6.883 | 4 | **0.142** | No |
| **Community Types** | 4.862 | 4 | **0.302** | No |
| Note: No significant differences were found between groups (p > 0.05). | | | | |

**Supplementary Table 3.** Regression Sensitivity Analysis.

| **Model Specification** | **Unstandardized Coeff. (B) for Env. Adaptation** | **Sig. (p)** | **Survey Mode Coeff. (B)** | **Survey Mode Sig. (p)** |
| --- | --- | --- | --- | --- |
| **Model A(Baseline)** | 0.865 | < .001 | -- | -- |
| **Model B (Controlled)** | 0.864 | < .001 | 0.016 | 0.686 |

**Supplementary Table 4.** Residential community samples selected in Guangzhou.

| **District**  **（11）** | **Low-Rent Housing Community** | **Commodity Housing Community** | **Affordable Housing Community** | **Resettlement Housing Community** | **Retirement Community** |
| --- | --- | --- | --- | --- | --- |
| **Yue Xiu** | Xingcheng | Mingyue Haoting | Wanke  Qinyue Court | Haiyue  Dong | Jiayi Elderly Care Home |
| **Hai Zhu** | Yue Wai Court | Zhujiang Dijingyuan | Jude Flower Court | Pazhou Xincun | Cixin Elderly Care Home |
| **Li Wan** | Dapeng Garden | Zhuguang Yujing | Fanghe Garden | Hesha Village Rehabilitation | Yingming Elderly Nursing Home |
| **Tian He** | Zhujiang  Jiayuan | Junjing Garden | Anxia Garden | Cuijing  Garden | Furui Xin Elderly Care Home |
| **Bai Yun** | Runhe Garden | Bitao Bay | Nanyue Huayuan | Chentian  Village | Changsong Aixin Elderly Care Service |
| **Huang Pu** | Luogang  He Yuan | Jinbi Century Garden | Tianlu Garden | Hetang Xia | Runkang Elderly Development |
| **Hua Du** | Songyuanli | Holiday Peninsula | Ziguang  Garden | Xinjie  Village | District Nursing Home |
| **Pan Yu** | Cheng Juan Yue Fu | Vanke Opal | Nanpu  Shidai | Huanan New Town | Taicheng Yiyuan Elderly Care Home |
| **Nan Sha** | Jia An Garden | Guangzhou Country Garden | Baoli Xingman  Garden | Nanyue  Mingzhu  Garden | District Nursing Home |
| **Cong Hua** | Jun Fu Xuan | Hopson Grand Joy Garden | Xiangyang  community | Hot Spring Lingnan Cultural Park | District Social Welfare Home |
| **Zeng Cheng** | Jinrui Garden | Fengya Garden | Huicui  Bay | Xiaofeng  Yunzhu | District Social Welfare Home |

（Source：The People's Government of Guangzhou Municipality（<https://www.gz.gov.cn/>）

Lianjia Guangzhou（<https://gz.lianjia.com/xiaoqu/>））
